# Racial and ethnic differences in COVID-19 infection and vaccine uptake across multiple waves of the pandemic in Southeast Michigan: a retrospective cohort study

**DOI:** 10.3389/fpubh.2025.1646801

**Published:** 2026-01-12

**Authors:** Elkhansa Sidahmed, Ramin Homayouni, Karen Childers, Chen Shen, Elie Mulhem

**Affiliations:** 1 Department of Family Medicine and Community Health, Oakland University William Beaumont School of Medicine, Rochester, MI, United States; 2 Corewell Health Research Institute, Grand, Rapids, MI, United States; 3 Department of Foundational Medical Studies, Oakland University William Beaumont School of Medicine, Rochester, MI, United States; 4 Henry Ford Health and Michigan State University Health Sciences, Detroit, MI, United States; 5 Department of Public Health Sciences, Henry Ford Health, Detroit, MI, United States

**Keywords:** COVID-19 infection rates, COVID-19 vaccine uptake, Hispanic or Latino, Middle Eastern or Arab, pandemic waves, racial and ethnic disparities

## Abstract

**Background:**

The COVID-19 pandemic revealed significant racial and ethnic disparities in the United States, yet limited data exist for Middle Eastern or Arab (MEA) ethnic group. We aimed to assess COVID-19 infection and vaccine rates among MEA and other racial and ethnic groups across multiple waves of the pandemic.

**Methods:**

We conducted a retrospective cohort study of adult patients who visited eight emergency departments (EDs) within a large Southeast Michigan healthcare system during the first 2 years of the pandemic. Five pandemic waves were defined: Initial (pre-October 2020), Holiday (October 2020–March 2021), Alpha (March 2021–June 2021), Delta (June 2021–December 2021), and Omicron (December 2021–June 2022). Chi-squared tests assessed infection differences, while logistic regression evaluated infection odds and Kaplan–Meier analysis for vaccine uptake.

**Results:**

Among 168,288 ED patients, 20,253 (12%) tested positive for SARS-CoV-2. MEA and Hispanic or Latino (HL) patients exhibited higher infection rates (19.1 and 20.9%, respectively) compared to Black and White patients (13.5 and 9.8%, respectively). MEA patients consistently had higher odds of infection across all waves, despite similar vaccination rates to White patients. Black and HL patients showed varying but higher likelihoods of infection across waves and lower vaccine uptake compared to White patients.

**Conclusion:**

MEA patients experienced disproportionately high infection rates in the ED despite comparable vaccination uptake to White patients. Black and HL individuals had both lower vaccine uptake and elevated infection risks. These disparities underscore the need for culturally tailored interventions to address health inequities in future pandemics.

## 1 Introduction

The COVID-19 pandemic, caused by the SARS-CoV-2 virus, emerged in Wuhan, China in late 2019 and quickly escalated into a global health crisis with significant increase in morbidity and mortality (1). The first laboratory-confirmed case in the United States was reported by the CDC in January 2020 (2). The state of Michigan reported its first case on March 10, 2020, coinciding with the World Health Organization’s (WHO) declaration of COVID-19 as a global pandemic and Michigan’s Governor declaring a state of emergency (3, 4). By April 2020, Michigan became the third state to exceed 20,000 confirmed COVID-19 cases, highlighting the rapid spread of the virus and severe impact of the virus on the state’s population (5).

COVID-19 progressed in a series of waves, each marked by varying infection rates and disease severity. In Michigan, over the initial two years of the pandemic, more than 1.8 million cases were documented across five distinct waves (6). The first two waves were dominated by the wild-type strain of SARS-CoV-2 and included the initial wave (before October 10, 2020) and the holiday wave (October 1, 2020–March 7, 2021) (7). The third wave (March 7, 2021–June 27, 2021) was characterized by the emergence of the Alpha variant (8). The overall infection rates decreased during this period, coinciding with the public availability of vaccines in May 2021 (9). The fourth wave, (June 27, 2021–December 19, 2021), was dominated by the Delta variant, which led to a resurgence in cases due to its heightened transmissibility and potential for severe illness (10, 11). The fifth wave (December 19, 2021–June 18, 2022) was attributed to the rise of the Omicron variant. Although Omicron variant had higher infection rates, it generally caused milder symptoms compared to previous variants, a trend likely influenced by the mutated virus, higher vaccination rates and growing herd immunity (12, 13).

Research has consistently shown that minority populations, including African Americans or Black and Hispanic or Latino (HL) individuals, have experienced a disproportionately high burden of COVID-19 infections and related adverse health outcomes (14–16). However, the impact of the pandemic on other minority groups, such as Middle Eastern or Arab (MEA) individuals, remains understudied (17). Additionally, there has been limited exploration of how infection rates and vaccination uptake differ across racial and ethnic groups throughout the different waves of the pandemic.

In Michigan, the pandemic exacerbated existing health disparities, disproportionately affecting Michigan’s diverse population, including Black and MEA populations (18–21). Therefore, understanding these disparities and the impact of vaccination on infection rates is crucial for informing public health strategies and preparing for future public health crisis (22).

This study aims to assess disparities in COVID-19 infection rates and vaccination uptake among Middle Eastern or Arab, Black, and Hispanic or Latino populations compared to Caucasians or White population in Southeast Michigan across multiple pandemic waves. By identifying patterns of infection and risk factors contributing to disparities, these findings will provide actionable insights into the effectiveness of public health measures and highlight areas where targeted interventions are needed. Such efforts are crucial for improving preparedness for future pandemics and addressing long-standing health inequities.

## 2 Methods

This is a retrospective study, which was reviewed by the Corewell Health East (CHE) Institutional Review Board (IRB) and granted an exempt classification with a waiver of informed consent. We followed the Strengthening the Reporting of Observational Studies in Epidemiology (STROBE) reporting guideline for cohort studies.

### 2.1 Study design and population

**Figure 1:**
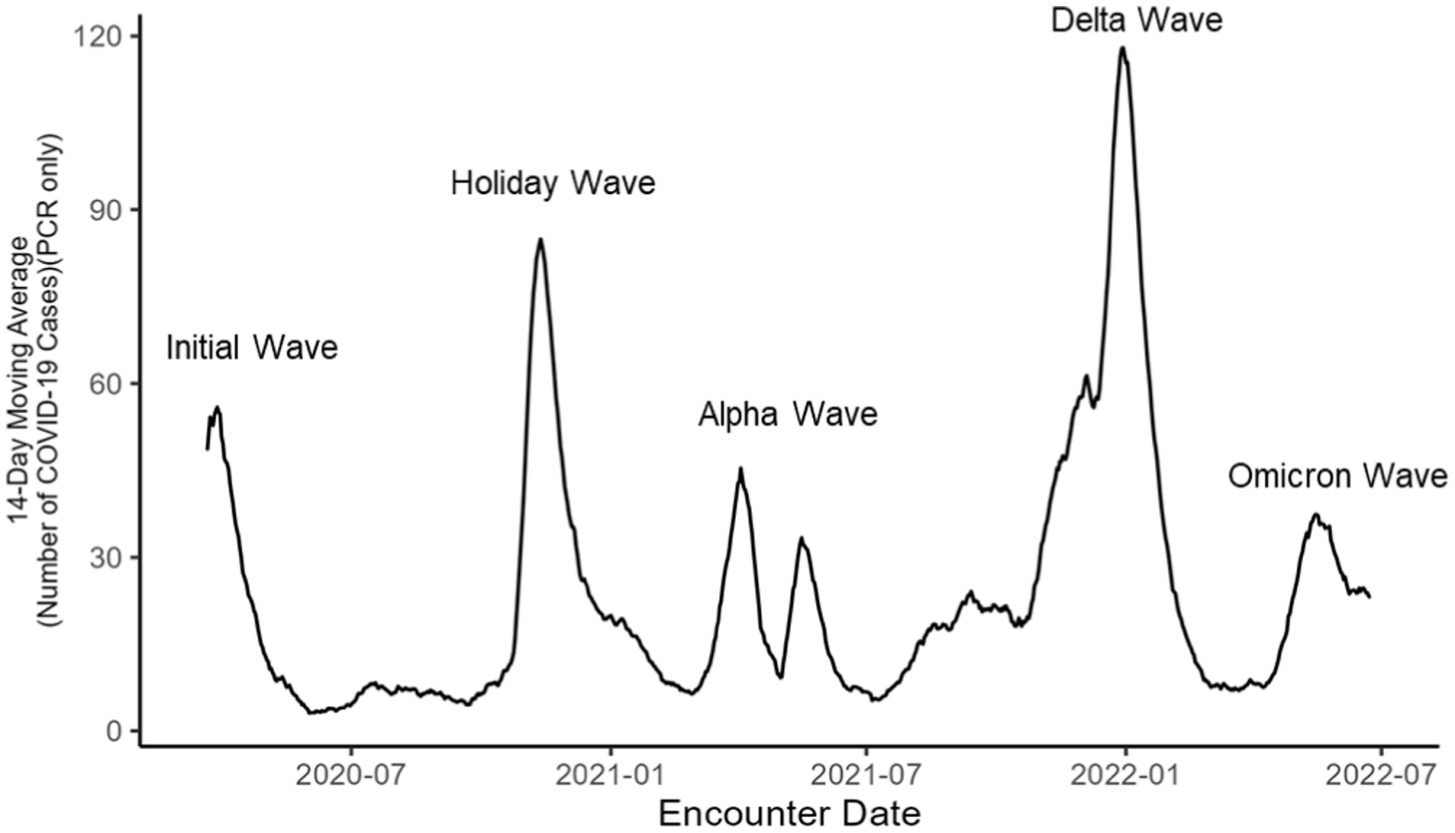
COVID-19 positive cases in southeast Michigan during the first 2 years of the pandemic.

The study included patients who visited one of the eight CHE emergency departments (EDs) March 1, 2020, to July 31, 2022. Eligible patients were 18 years and older who were tested for SARS-CoV-2 by nasopharyngeal qualitative polymerase chain-reaction (PCR) or antigen tests during their ED visit. During the study period, the COVID testing policy in the ED shifted from testing only symptomatic patients to testing all patients. The study included all individuals who were tested for COVID, regardless of whether they had symptoms. To manage multiple records during the study periods, a maximum of one record per wave was retained. If a patient tested positive within a wave, the first positive test result was retained. If no positive test occurred during a given wave, then the first negative test result was retained. During the study period, five waves of COVID-19 were identified using data from CHE, attributable to different SARS-CoV-2 variants: the wild type of variant, which includes both the initial wave, and the holiday; the alpha variant wave; the delta variant wave; and the omicron variant wave (Figure 1).

### 2.2 Data sources

Data were extracted from electronic medical records (EMR) and combined with immunization record from Michigan Care Improvement Registry (MCIR). The dataset included demographic such as, age, sex, race and ethnicity, BMI, and insurance type at the time of the ED encounter. Clinical data included problem list and medical history over a five-year look-back period from the first (index) ED encounter. Chronic conditions were identified using ICD-10 codes as defined by the Centers for Medicare & Medicaid Services (CMS) Chronic Conditions Data Warehouse 2021. Immunization status was determined according to CDC guidelines and defined as either fully or partially vaccinated. Vaccination data were obtained from the same ED cohort (linked via EMR to Michigan vaccine registry (MCIR), not the general population, allowing direct comparison. An individual was considered fully vaccinated 2 weeks after receiving the second dose of the BNT162b2 or mRNA-1273 vaccine or 2 weeks after a single dose of the Ad26.COV2.S vaccine. Also, an individual was considered partially vaccinated if they received one dose of a multi-dose COVID-19 vaccine and did not receive the second dose by the end of the study or by the end of each wave when running models for each wave separately. An individual was categorized as unvaccinated if did not receive any type of COVID-19 vaccine.2.3. Vaccination status was defined as the cumulative status by the end of each respective wave for the wave-specific analyses. For the aggregated analysis across all waves, vaccination status reflected the most recent vaccination record available through the end of the study period or at the last ED visit within each wave, whichever occurred first.

### 2.3 Outcomes and exposures

The primary outcome was SARS-CoV-2 positive lab test. The secondary outcome was vaccine status. The primary exposures of interest were race and ethnicity, documented separately in the electronic medical records (EMR). Patients were categorized as Caucasian or White race and non-Hispanic ethnicity (hereafter White), African American or Black (hereafter Black), Hispanic or Latino (HL), and Middle Eastern or Arab (MEA) ethnicity. To differentiate MEA ethnicity from White race of European origin, patients who chose White race and MEA ethnicity were removed from the White group, ensuring that all patients in the study had only one race or ethnicity designation.

### 2.4 Statistical analysis

The demographic and clinical characteristics of patients who tested positive for SARS-CoV-2 were compared across different racial and ethnic groups, including White, Black, Hispanic or Latino (HL), and Middle Eastern or Arab (MEA). Summary statistics were calculated using the Kruskal-Wallis rank sum test for continuous variables and Pearson’s chi-square test for categorical variables. Kaplan–Meier plots were used to illustrate the difference in COVID-19 vaccine trends (fully vaccinated and partially vaccinated), stratified by race and ethnicity between January 1, 2021, and July 31, 2022. A multivariable logistic regression model was employed to determine the odds of receiving a positive COVID-19 test by race and ethnicity group. The model included the coefficients of wave, race/ethnic group, the wave*group interaction, age, and covariates indicating the presence the following comorbidities: cancer, atrial fibrillation, hyperlipidemia, hypertension, ischemic heart disease, rheumatoid arthritis, and stroke/ Transient Ischemic Attack. A type III test for the wave*group interaction term, which assesses the significance of the interaction term after accounting for all other effects in the model, was statistically significant (*p* < 0.0001). Odds ratios were calculated comparing the odds of a positive COVID-19 test for each race/ethnicity compared to White patients with a Dunnett adjustment for multiple comparisons. Sample size of 168,288 was determined to provide at least 80% power to detect adjusted odds ratio differences > 1.2. All statistical tests were two-sided and *p*-values of less than 0.05 were considered statistically significant. All analyses were performed using R (version 4.2.2; R Foundation for Statistical Computing) or SAS (version 9.4; SAS Institute, Inc., Cary, NC).

## 3 Results

### 3.1 Study population

A total of 168,288 patients aged ≥18 years who visited one of the emergency departments (EDs) at Corewell Health East and were tested for SARS-CoV-2 (Supplementary Table S1). Among these 19,809 patients (11.8%) tested positive for the virus (Table 1). There was a significant (*p* < 0.001) difference in positivity between the different racial or ethnic groups (Table 1). Specifically, the highest positivity was observed among Hispanic or Latino (HL; 20.9%), followed by Middle Eastern or Arab (MEA; 19.1%) and Black patients (13.5%), compared to White patients (9.8%).

**Table 1:** Characteristics of patients who tested positive for SARS-CoV-2 in the ER across all COVID-19 waves by race or ethnicity.

Characteristics	Overall	White	MEA	Black	Hispanic or Latino	*p*-value
*n*	19,809	9,876	1838	7,158	937	
Gender, Male	9,231 (46.6)	4,760 (48.2)	1,018 (55.4)	2,899 (40.5)	431 (46.0)	<0.001
Age, year						<0.001
18–34	4,450 (22.5)	1,380 (14.0)	393 (21.4)	2,423 (33.9)	254 (27.1)	
35–50	4,249 (21.4)	1,639 (16.6)	455 (24.8)	1844 (25.8)	311 (33.2)	
51–64	4,614 (23.3)	2,390 (24.2)	426 (23.2)	1,602 (22.4)	196 (20.9)	
65–74	3,028 (15.3)	1881 (19.0)	283 (15.4)	761 (10.6)	103 (11.0)	
75+	3,468 (17.5)	2,586 (26.2)	281 (15.3)	528 (7.4)	73 (7.8)	
Obese	8,541 (48.2)	4,095 (44.9)	644 (41.3)	3,390 (54.4)	412 (51.4)	<0.001
Medicaid	5,422 (28.9)	1,419 (15.1)	840 (47.1)	2,807 (41.6)	356 (43.2)	<0.001
Chronic condition present						
Asthma	3,814 (19.3)	1806 (18.3)	254 (13.8)	1,619 (22.6)	135 (14.4)	<0.001
Atrial fibrillation	2,282 (11.5)	1,659 (16.8)	183 (10.0)	402 (5.6)	38 (4.1)	<0.001
Cancer	1,467 (7.4)	1,040 (10.5)	80 (4.4)	311 (4.3)	36 (3.8)	<0.001
Chronic kidney disease	6,613 (33.4)	3,821 (38.7)	574 (31.2)	2008 (28.1)	210 (22.4)	<0.001
Chronic obstructive pulmonary disease	4,587 (23.2)	2,812 (28.5)	308 (16.8)	1,347 (18.8)	120 (12.8)	<0.001
Depression	4,654 (23.5)	2,870 (29.1)	241 (13.1)	1,356 (18.9)	187 (20.0)	<0.001
Diabetes	6,276 (31.7)	3,352 (33.9)	690 (37.5)	1980 (27.7)	254 (27.1)	<0.001
Heart Failure	3,208 (16.2)	1979 (20.0)	264 (14.4)	896 (12.5)	69 (7.4)	<0.001
Hyperlipidemia	9,081 (45.8)	5,550 (56.2)	929 (50.5)	2,291 (32.0)	311 (33.2)	<0.001
Hypertension	10,877 (54.9)	6,097 (61.7)	889 (48.4)	3,536 (49.4)	355 (37.9)	<0.001
Ischemic heart disease	4,877 (24.6)	3,103 (31.4)	455 (24.8)	1,199 (16.8)	120 (12.8)	<0.001
Rheumatoid arthritis	6,192 (31.3)	3,967 (40.2)	487 (26.5)	1,573 (22.0)	165 (17.6)	<0.001
Stroke/transient ischemic attack	2,418 (12.2)	1,478 (15.0)	157 (8.5)	713 (10.0)	70 (7.5)	<0.001
Vaccination status						
Fully vaccinated	3,011 (15.2)	2044 (20.7)	160 (8.7)	766 (10.7)	63 (6.7)	<0.001
Only one dose	3,625 (18.3)	2,429 (24.6)	188 (10.2)	952 (13.3)	77 (8.2)	<0.001
Unvaccinated	13,173 (66.5)	5,403 (54.7)	1,490 (81.1)	5,440 (76.0)	797 (85.1)	<0.001

Data presented in numbers *n*, and percentage (%). *p-value shown is the result of the Pearson chi-square test of association. MEA, Middle Eastern or Arab.

### 3.2 Demographic and clinical characteristics of COVID-19 positive individuals

Compared to White patients, all other racial or ethnic groups who tested positive for COVID-19 in the ED were generally younger (Table 1). The proportion of positive cases for age categories 18–34 and 35–50 combined, were highest in HL (60.3%) patients, followed by Black (59.7%), MEA (46.2%), and White (30.0%) patients. Conversely, older patients (65–74 and 75+) had the highest proportion of positive cases (45.2%) among White patients followed by MEA (30.7%), Black (18%), and HL (18.8%) patients. Among those who tested positive, Medicaid beneficiaries were highest proportion among MEA patients (47.1%), followed by HL (43.2%), Black (41.6%) and White (15.1%) patients. Obesity was more prevalent among Black (54.4%) and HL (51.4%) patients compared to White (44.9%) and MEA (41.3%) patients. In addition, White patients had higher proportions of chronic kidney disease (38.7%), depression (29.1%), and hyperlipidemia (56.2%). Black patients had a higher proportion of asthma (22.6%), while MEA patients had a higher proportion of diabetes (37.5%). Similar demographic and clinical characteristics were observed among the base population of patients who visited the ED and were tested positive for the SARS-CoV-2 virus (Supplementary Table S1).

### 3.3 Positivity and vaccination trends across pandemic waves

**Figure 2:**
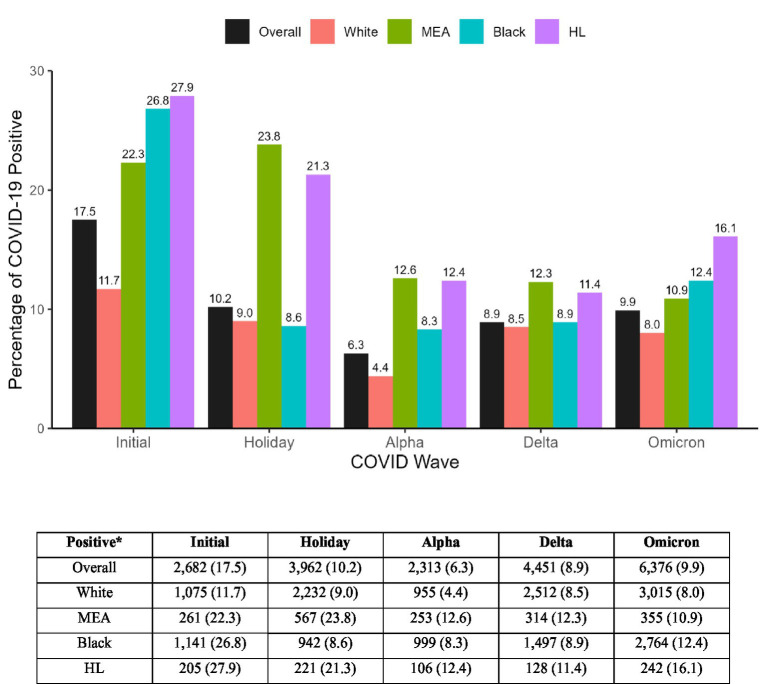
The proportion of COVID-19 positive individuals in each race or ethnicity by wave. *Data presented in numbers *n* and percentage (%). HL, Hispanic or Lattino; MEA, Middle Eastern or Arab.

In the initial wave of the pandemic, the proportion of COVID-19 positive individuals for all minority patients were higher compared to White patients (Figure 2). Also, the proportion of COVID-19 positive patients within each race was highest in the initial wave compared to other waves (Figure 2). Comparing the holiday wave to the initial wave, infection rates among Black patients dramatically decreased from 26.8 to 8.6% but remained similar for MEA (22.3 to 23.8%), HL (27.9 to 21.3%) and White patients (11.7 to 9.0%). In later waves (Alpha, Delta and Omicron), all minority groups exhibited higher infection rates compared to White patients, but to varying levels (Figure 2).

### 3.4 Differences in COVID-19 vaccination rates between racial and ethnic groups

**Figure 3:**
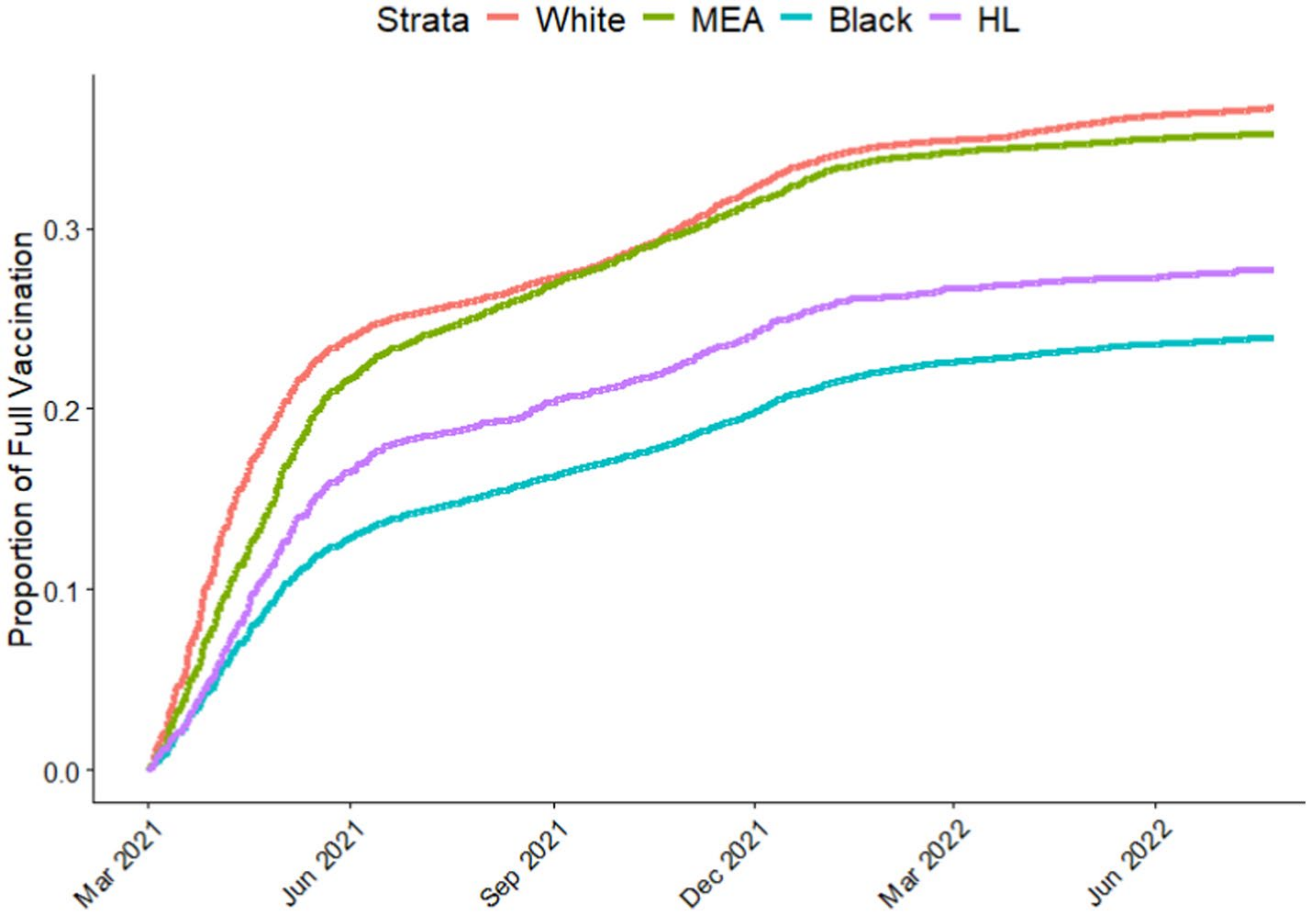
Proportion of individuals in each race or ethnicity who received two doses of COVID-19 vaccine. HL, Hispanic or Lattino; MEA, Middle Eastern or Arab.

To investigate if the differences in infection rates between racial and ethnic groups were due to differences in vaccination, we analyzed vaccination data aggregated from the EMR and state records. Both fully vaccinated (Figure 3) and partially vaccinated (Figure S1). We included the vaccine data from March 2021 (during the Alpha wave) when became publicly available to the general population. Plots indicated that White and MEA groups had higher vaccination rates compared to Black and HL. Initially, White and MEA groups showed quicker increases in vaccination rates compared to Black and HL groups. Notably, the vaccination curve for MEA patients closely followed or slightly exceeded that of White patients. Black patients consistently exhibited the lowest vaccination rate throughout the study period.

### 3.5 Differences in COVID-19 infection by racial and ethnic groups for all five waves

**Figure 4:**
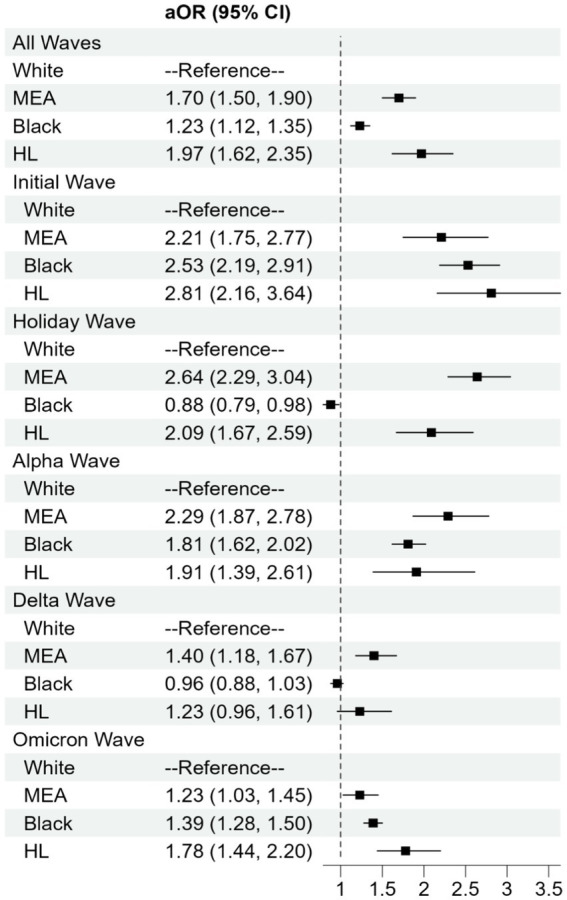
Adjusted odds ratios (aOR) and 95% CI for COVID-19 positivity by race or ethnicity during different waves of the pandemic. Regression models were adjusted for age, gender, obesity, insurance coverage and co-morbidities. HL, Hispanic or Lattino; MEA, Middle Eastern or Arab.

Using aggregated data across all pandemic waves, multivariable logistic regression showed significantly higher odds of testing positive for COVID-19 in all minority groups compared to White patients after adjusting for demographic, and clinical variables. The adjusted odds ratios and [95% confidence intervals] for testing positive for COVID-19 were 1.70 [1.50, 1.90] for MEA, 1.23 [1.12, 1.35] for Black and 1.97 [1.62, 2.35] for HL patients compared to White patients (Figure 4).

Multivariable regression analysis of each wave separately showed that the odds of infection varied between minority groups during different pandemic waves. During the initial wave, all minority groups had significantly higher odds of testing positive for COVID-19 compared to White patients, with adjusted odds ratios [95% confidence intervals] of 2.23 [1.77–2.81] for MEA, 2.55 [2.22–2.95] for Black, and 2.84 [2.18–3.69] for HL patients.

During the holiday wave, MEA and HL patients had an even higher aOR of 2.67 [2.31–3.08] and 2.11 [1.69–2.63], respectively, while Black patients showed a significantly lower aOR of 0.88 [0.78–0.98] compared to White patients. In Alpha and Omicron waves, all minority groups had significantly higher odds of testing positive compared to White group. During the Delta wave, only MEA patients had a significantly higher odds 1.42 ([1.19, 1.69]) of testing positive compared to White patients, while the odds of testing positive was 0.95 (95% CI: 0.86, 1.04) for Black and 1.24 ([95% CI: 0.94, 1.63]) for HL. A sensitivity analysis incorporating the emergency department site as a random effect yielded results consistent with the primary analysis, confirming that the observed disparities were not driven by site-specific clustering.

## 4 Discussion

To our knowledge, this is the first study to comprehensively assess COVID-19 positivity during distinct pandemic waves while considering vaccination status across different racial and ethnic groups, including Middle Eastern or Arab (MEA) individuals. White patients consistently had the lowest infection rates, even in the early waves of the pandemic when vaccines were not available. In addition, White patients had the highest vaccination rates compared to all other racial or ethnic groups. Our findings align with an established body of research that report significant racial and ethnic disparities in COVID-19 infection rates, hospitalization, and mortality (23). Additionally, several studies have shown that minority communities, particularly Hispanic/Latino (HL), Black, and MEA populations, were disproportionately affected by the pandemic, even when vaccination rates were not considered (18, 21, 24–26). Our findings align with a well-established body of research reporting significant racial and ethnic disparities in COVID-19 infection rates, hospitalizations, and mortality (23). Our analysis during the initial wave (prior to vaccine availability) revealed significantly higher odds of testing positive for COVID-19 among all minoritized groups compared to White patients. These disparities were largely driven by higher prevalence of chronic conditions (e.g., diabetes, hypertension) and social determinants of health, including limited access to healthcare, and increased exposure through essential work and multigenerational housing (27). Moreover, similar findings were reported from a retrospective cohort study of adult patients from diverse racial and ethnic backgrounds, tested for COVID-19 across 155 emergency departments during the early (March–June 2020) and late (July–September 2020) phases of the pandemic. Although that study phase definitions differ slightly from ours (initial wave defined as pre-October 2020), the timeframes largely overlap. This study found significantly higher adjusted odds of testing positive for COVID-19 among minoritized groups, including Hispanic/Latino and Black. Our analysis similarly revealed elevated odds of infection among these populations, highlighting persistent disparities in COVID-19 exposure and outcomes during the early stages of the pandemic (28). MEA patients who visited the ED, consistently showed high positivity rates throughout all pandemic waves compared to White patients, despite having similar vaccination rates. The similarity in vaccination rates could be attributed to the strong emphasis on vaccine awareness and disease prevention within the MEA communities (29, 30). Community leaders and healthcare providers may have effectively promoted vaccination within the MEA community (31, 32). Additionally, MEA individuals may have had better access to healthcare facilities and vaccination sites compared to other minority groups (33). However, MEA patients who presented to the ED experienced higher COVID-19 positivity rates throughout the pandemic, suggesting that factors beyond vaccination contributed to higher infection rates among this group. Several factors may have contributed to higher positivity rates among MEA. First, MEA population in this region are generally employed as essential workers and in services industries that had higher exposure to the virus. Second, factors such as multigenerational households, crowded living conditions and large family gatherings may have facilitated the spread of the virus (25). Third, lower socioeconomic status and limited access to healthcare services may have further exacerbated infection rates (34).

Our study shows that, compared to White patients, HL patients had consistently higher positivity rates across all pandemic waves and had lower vaccination rates. These findings are consistent with existing literature documenting disproportionate impact of COVID-19 on Hispanic communities (35, 36). Similar to MEA, Hispanic individuals work in essential jobs that require physical presence, increasing their risk of exposure (37). Many Hispanic families live in multigenerational households or crowded housing, which also facilitates virus transmission (38, 39). Furthermore, previous studies have shown that social determinants such as employment in essential services and lack of access to healthcare contribute significantly to higher infection rates among minority groups (15, 17). For example, limited access to healthcare and language barriers can delay testing and treatment, leading to higher infection rates (40). The lower vaccination rates in HL may be due to vaccine hesitancy and a lack of knowledge about vaccine efficacy and safety (41). Access barriers, such as difficulties in transportation, inflexible work schedules, fear of deportation for undocumented immigrants and language barriers, also likely played a role in hindering vaccination efforts (42, 43).

COVID-19 positivity rates for Black patients fluctuated across different pandemic waves. In addition, Black patients had the lowest vaccination rates compared to all other racial or ethnic groups. During the initial wave, higher infection rates among Black individuals could be attributed to increased exposure and a lack of adherence to public health measures, possibly due to the newness of the virus and limited information about its transmission. During the Holiday wave, the infection rate among the Black population were similar to the White population. Our findings were similar to previous studies that found non-Hispanic Black had lower infection rates during the Holiday surge compared to the initial wave (44). Additionally, a previous report showed that while Black and HL generally had higher overall infection rates than White individuals, only Black individuals had similar rates to White individuals during the Holiday and Delta waves (45).

The reduction in COVID-19 infection among Black individuals may be due to higher mortality in the initial wave which lead to enhanced fear among black community, resulting in social isolation and other mitigation efforts during the Holiday wave. For example, one study found Black individuals, after experiencing higher infection and mortality early in the pandemic, were less likely to have engaged in risky holiday gatherings in late 2020 (46). Furthermore, the lower COVID-19 infection rates in some Black communities during the holiday wave were likely due to targeted vaccination efforts by Black churches. For example, the National Black Church Initiative (NBCI) launched a campaign in early 2021, partnering with the CDC, mobilizing 150,000 churches, and hosting over 150 vaccination events during high-risk periods, including holidays (47). However, our results suggest that these protective effects may have been short-lived since Black patients were significantly more likely to be infected than White patients in subsequent pandemic waves (48).

In this study we observed Black patients had the lowest vaccination rates compared to all other racial or ethnic groups. This finding suggests a significant vaccine hesitancy, possibly due to historical mistrust of the healthcare system (49). Misinformation about vaccine safety and efficacy may also deter individuals from getting vaccinated. Additionally, barriers such as lack of transportation, inflexible work schedules, and limited access to healthcare facilities further hinder vaccination efforts. Taken together, our results highlight the complex interplay of factors that contributed to variation in infection rates among Black, such as the emergence of new variants, public health measures, and vaccination uptake.

A major strength of this study is the large cohort size, which included different racial and ethnic groups, including MEA as well as the comprehensive analysis of infection and vaccination rates across multiple pandemic waves. However, the study has some limitations. The study is retrospective and relies on the accuracy of electronic medical records, which may have inaccuracies or missing information. Additionally, the categorization of race and ethnicity is self-reported, which may not capture the full diversity within these groups, especially for MEA and HL categories. Also, the study population was restricted to patients presenting to the emergency department, excluding individuals with milder COVID-19 symptoms remained home or sought outpatient care. As this study was conducted in Southeast Michigan, the findings may not be directly transferable to regions with distinct demographic characteristics or generalized to other regions.

In conclusion, our study highlights the significance of the racial and ethnic disparities in COVID-19 infection rates and vaccination uptake in Southeast Michigan. Hispanic and MEA individuals experienced higher infection rates across most pandemic waves, while Black individuals had a variable rate in different waves. This study emphasizes the need for further investigation into factors that affected infection rates other than vaccine uptake in these racial and ethnic groups in Southeast Michigan. Understanding these drivers is essential for designing targeted public health messaging and interventions. For future epidemics or pandemics, especially in Michigan, it is important to strengthen community partnerships and engage trusted local organizations, such as Black churches early in the response. Broader efforts should include collecting real-time, disaggregated data by race, ethnicity, geography, and socioeconomic status to guide equitable interventions. Also, expanding access to care, ensuring equitable distribution of testing, treatment, and vaccines. Moreover, a social determinant of health and improving vaccine outreach are crucial steps towards reducing these disparities and improving health outcomes in vulnerable populations.

## Data Availability

The original contributions presented in the study are included in the article/Supplementary material, further inquiries can be directed to the corresponding author/s.
