# Supplementary material for: Racial and ethnic differences in COVID-19 infection and vaccine uptake across multiple waves of the pandemic in Southeast Michigan: a retrospective cohort study
Source: Front Public Health. 2026 Jan 12;13:1646801. doi: 10.3389/fpubh.2025.1646801 (PMC12832436; doi:10.3389/fpubh.2025.1646801)
Supplement: Supplementary file 1 [file Supplementary_file_1.docx]

**Supplementary Figures and Tables**

**Supplementary Table 1.** Characteristics of patients who visited the emergency department between March 1, 2020, to July 31, 2022, and were tested for SARS-CoV-2 at any of the eight Corewell Health hospitals in metro Detroit.

|  | Overall | White | MEA | Black | Hispanic or Latino | p-value |
| --- | --- | --- | --- | --- | --- | --- |
| n | 168,288 | 101,268 | 9,610 | 52,937 | 4,473 |  |
| Gender, Male | 74,535 (44.3) | 46,694 (46.1) | 4,889 (50.9) | 21,011 (39.7) | 1,941 (43.4) | <0.001 |
| Age, year |  |  |  |  |  | <0.001 |
| 18-34 | 35,935 (21.4) | 15,339 (15.1) | 2,576 (26.8) | 1,6512 (31.2) | 1,508 (33.7) |  |
| 35-50 | 32,272 (19.2) | 16,459 (16.3) | 2,165 (22.5) | 1,2400 (23.4) | 1,248 (27.9) |  |
| 51-64 | 37,358 (22.2) | 23,097 (22.8) | 1,928 (20.1) | 11,450 (21.6) | 883 (19.7) |  |
| 65-74 | 27,779 (16.5) | 19,227 (19.0) | 1,346 (14.0) | 6,780 (12.8) | 426 (9.5) |  |
| 75+ | 34,944 (20.8) | 27,146 (26.8) | 1,595 (16.6) | 5,795 (10.9) | 408 (9.1) |  |
| Obese | 66,612 (42.1) | 37,785 (39.1) | 3,290 (38.4) | 23,690 (48.5) | 1,847 (46.3) | <0.001 |
| Medicaid | 40,986 (25.4) | 15,061 (15.5) | 4,331 (46.5) | 19,913 (39.3) | 1,681 (41.5) | <0.001 |
| Chronic Condition Present |  |  |  |  |  |  |
| Cancer | 16,307 (9.7) | 12,228 (12.1) | 593 (6.2) | 3,298 (6.2) | 188 (4.2) | <0.001 |
| Asthma | 31,633 (18.8) | 17,962 (17.7) | 1,301 (13.5) | 11,651 (22.0) | 719 (16.1) | <0.001 |
| Atrial Fibrillation | 21,465 (12.8) | 16,657 (16.4) | 979 (10.2) | 3,609 (6.8) | 220 (4.9) | <0.001 |
| Chronic Kidney Disease | 55,419 (32.9) | 35,844 (35.4) | 2,878 (29.9) | 15,663 (29.6) | 1,034 (23.1) | <0.001 |
| Chronic Obstructive Pulmonary Disease | 39,106 (23.2) | 26,710 (26.4) | 1,631 (17.0) | 10,196 (19.3) | 569 (12.7) | <0.001 |
| Depression | 44,289 (26.3) | 31,040 (30.7) | 1,548 (16.1) | 10,630 (20.1) | 1,071 (23.9) | <0.001 |
| Diabetes | 48,798 (29.0) | 29,456 (29.1) | 3,359 (35.0) | 14,888 (28.1) | 1,095 (24.5) | <0.001 |
| Heart Failure | 27,484 (16.3) | 18,191 (18.0) | 1,366 (14.2) | 7,549 (14.3) | 378 (8.5) | <0.001 |
| Hyperlipidemia | 79,069 (47.0) | 54,731 (54.0) | 4,767 (49.6) | 18,087 (34.2) | 1,484 (33.2) | <0.001 |
| Hypertension | 94,384 (56.1) | 60,840 (60.1) | 4,573 (47.6) | 27,277 (51.5) | 1,694 (37.9) | <0.001 |
| Ischemic Heart Disease | 44,439 (26.4) | 31,124 (30.7) | 2,354 (24.5) | 10,294 (19.4) | 667 (14.9) | <0.001 |
| Rheumatoid Arthritis | 55,695 (33.1) | 39,930 (39.4) | 2,557 (26.6) | 12,348 (23.3) | 860 (19.2) | <0.001 |
| Stroke/Transient Ischemic Attack | 22,127 (13.1) | 14,750 (14.6) | 975 (10.1) | 6,069 (11.5) | 333 (7.4) | <0.001 |
| Vaccination Status |  |  |  |  |  |  |
| Fully Vaccinated | 51,521 (30.6) | 35,929 (35.5) | 2,849 (29.6) | 11,711 (22.1) | 1,032 (23.1) | <0.001 |
| At least one dose | 59,066 (35.1) | 37,526 (37.1) | 3,046 (31.7) | 12,618 (23.8) | 1,104 (24.7) | <0.001 |
| COVID-19 Test Results |  |  |  |  |  |  |
| Positive | 19,809 (11.8) | 9,876 (9.8) | 1,838 (19.1) | 7,158 (13.5) | 937 (20.9) | <0.001 |

Results presented in numbers n, and percentage (%).

*P-value shown is the result of the Pearson chi-square test of association. MEA: Middle Eastern or Arab

**
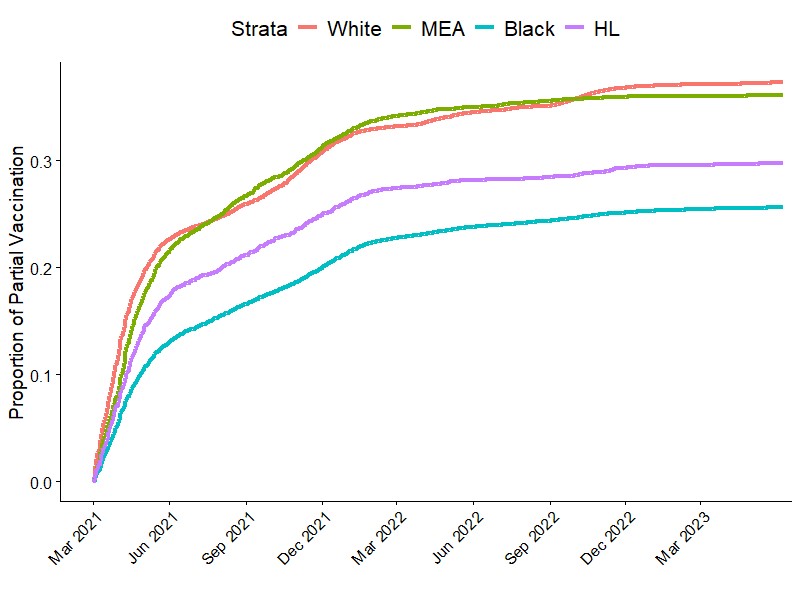
**

**Supplementary Figure 1. Uptake of at least one dose of vaccine.**

HL: Hispanic/Lattino, MEA: Middle Eastern or Arab
